# Charge Photogeneration and Recombination in Fluorine-Substituted Polymer Solar Cells

**DOI:** 10.3389/fchem.2022.846898

**Published:** 2022-02-24

**Authors:** Rong Hu, Yurong Liu, Jun Peng, Jianjun Jiang, Mengyao Qing, Xiaochuan He, Ming-Ming Huo, Wei Zhang

**Affiliations:** ^1^ School of Materials Science and Engineering, Chongqing University of Arts and Sciences, Chongqing, China; ^2^ School of Physics and Materials Science, Guangzhou University, Guangzhou, China; ^3^ Songshan Lake Materials Laboratory, Dongguan, China; ^4^ Qingdao Branch, Naval Aeronautical University, Qingdao, China; ^5^ Research Center for Advanced Information Materials (CAIM), Huangpu Research and Graduate School of Guangzhou University, Guangzhou, China; ^6^ Guangzhou University-Linköping University Research Center on Urban Sustainable Development, Guangzhou University, Guangzhou, China

**Keywords:** polymer solar cells, fluorine substitution, charge transport, charge recombination, power conversion efficiency

## Abstract

In this contribution, we studied the effect of fluorine substitution on photogenerated charge generation, transport, and recombination in polymer solar cells. Two conjugated polymer materials, PBDTTT-E (fluorine free) and PTB7 (one fluorine substitution), were compared thoroughly. Meanwhile, various characterization techniques, including atomic force microscopy, steady-state spectroscopy, transient absorption spectroscopy, spectroelectrochemistry, and electrical measurements, were employed to analyse the correlation between molecular structure and device performance. The results showed that the influence of fluorine substitution on both the exciton binding energy of the polymer and the carrier recombination dynamics in the ultrafast timescale on the polymer was weak. However, we found that the fluorine substitution could enhance the exciton lifetime in neat polymer film, and it also could increase the mobility of photogenerated charge. Moreover, it was found that the SOMO energy level distribution of the donor in a PTB7:PC71BM solar cell could facilitate hole transport from the donor/acceptor interface to the inner of the donor phase, showing a better advantage than the PBDTTT-E:PC71BM solar cell. Therefore, fluorine substitution played a critical role for high-efficiency polymer solar cells.

## Introduction

In the field of solar energy, polymer solar cells (PSCs) have attracted much attention owing to their flexibility, low cost, light weight, material diversification, and large-area solution processing (Kim et al., 2007; Arias et al., 2010; Lu et al., 2018). Recently, the photovoltaic systems of halogenated polymers represented by PM6 or D18 blended with non-fullerene have achieved the power conversion efficiency (PCE) of 16%–18% (Lin et al., 2020; Liu et al., 2020; Li C. et al., 2021), even up to 19.6% (Wang et al., 2021), showing a bright application prospect.

Fluorine (F) substitution is an important method to adjust the conformation, optical, and electrical properties of polymers (Dunitz and Taylor, 1997; Jackson et al., 2013; Zhang et al., 2014; Zhang et al., 2017; Chao et al., 2018; Dehnen et al., 2021) owing to its great electronegativity and strong electron-withdrawing property. Hence, it is usually adopted as a substituent to substitute H atom on benzene (or thiophene) rings, so as to adjust the energy levels and optical absorption of a polymer in the active layer. For example, it was found that the highest occupied molecular orbital (HOMO) energy level of polymers could be shifted down by halogen substitution (Chen H. et al., 2018; Liang et al., 2010). Besides, the substitution position and the number of fluorine elements in conjugated polymer or in non-fullerene also showed a significant impact on the coplanarity of molecules, which could further determine the morphology (such as crystallinity, domain size, phase separation scale, and network interpenetrating structure) of bulk heterojunction (Jia et al., 2019; Chen F. X. et al., 2018). As known, the photophysical processes in the PSC device, such as exciton diffusion and dissociation, charge transfer, charge generation, charge transport, charge recombination, and charge collection, are closely related to the morphology of the active layer (Clarke et al., 2009; Po et al., 2010; Zhang et al., 2012). Accordingly, fluorine substitution would play an important role in the photophysical process and, consequently, the performance of polymer solar cells. The ultrafast photoelectric conversion processes in PSCs that are based on fluorine-substituted conjugated polymers (such as PTB7-Th, PffBT4T-2OD, and PBDBT-2F) have been studied extensively by using time-resolved spectroscopy and transient photoelectric measurements (Lu and Yu, 2014; Wu et al., 2018; Zhang et al., 2019; Wang et al., 2020). Also, most of the contributions focused on the photoelectric conversion mechanism that F-substituted polymers matched with different acceptors (Moritomo et al., 2016; Sharma et al., 2016; Sharma et al., 2018; Matheson et al., 2019; Liu et al., 2021), or morphology regulation of F-substituted polymer-based active layers (He et al., 2014; Kniepert et al., 2015; Chen L. et al., 2018). However, the targeted research about the influence of fluorine atoms on the photoelectric conversion process is still unclear yet for fluorine-substituted polymers.

**FIGURE 1:**
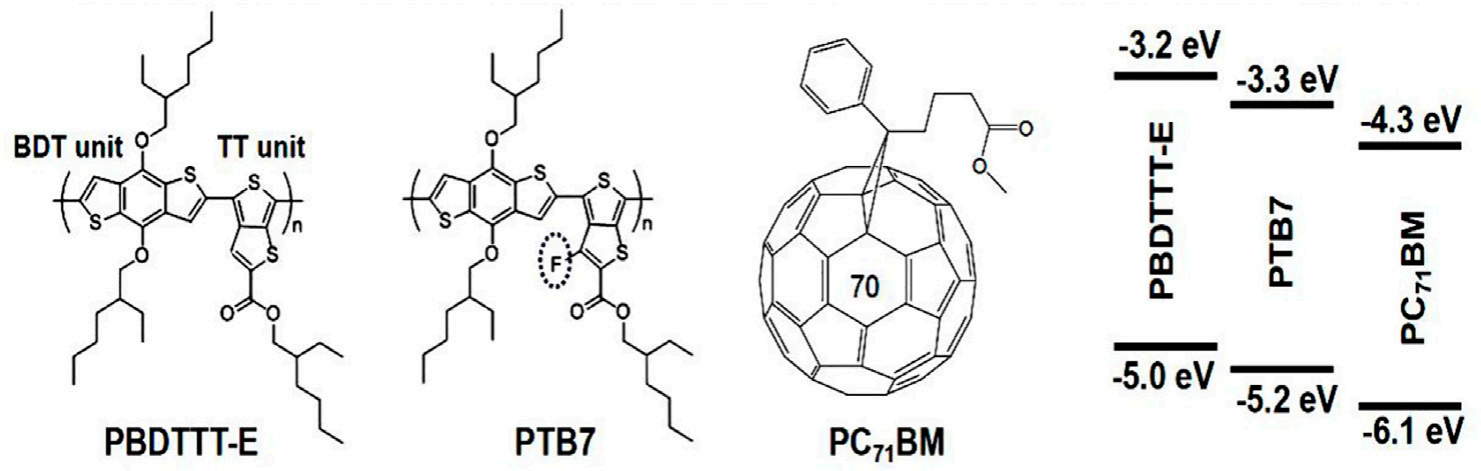
Chemical structures of PBDTTT-E, PTB7, and PC_71_BM and their energy levels.

In this contribution, for targeted study of the effect of fluorine substitution on photogenerated charge generation, transport, and recombination in PSCs, we consciously employed poly(thieno(3,4-b)-thiophene/benzodithiophene (PTB7) and poly(4,8-bis-substituted-benzo(1,2-b:4,5-b′)dithiophene-2,6-diyl-alt-4-substituted-thieno(3,4-b)thiophene-2,6-diyl) (PBDTTT-E) as comparative research objects. Their difference is the presence or absence of a fluorine atom substitution on the thienothiophene (TT) unit (Figure 1). Multiple characterization methods, including atomic force microscopy (AFM), steady-state spectroscopy, transient absorption spectroscopy (TAS), spectroelectrochemistry (SEC), and electrical measurements, were conducted to reveal the relationship between molecular structure and device performance. The results showed that fluorine substitution had an insignificant effect on polymer exciton binding energy and carrier recombination dynamics in the ultrafast timescale. Nevertheless, it was found that fluorine substitution could enhance the lifetime of polymer exciton, and it also could increase the mobility of photogenerated charge. Moreover, we found that the singly occupied molecular orbital (SOMO) energy level distribution of the donor in the PTB7:PC71BM solar cell could facilitate hole transport from the donor/acceptor (D/A) interface to the inner of the donor phase, showing a better advantage than the PBDTTT-E:PC71BM solar cell. Thus, the longer exciton lifetime and appropriate energy level arrangement in the PTB7:PC71BM device made the PCE higher than that of the PBDTTT-E:PC71BM device.

## Materials and Methods

### Fabrication of PSCs

In this work, the photovoltaic materials, PBDTTT-E (*M*_w_>40,000, PDI: 1.8–2.0), PTB7 (*M*_w_>40,000, PDI: 1.8–2.0), and (6,6)-phenyl-C71-butyric acid methyl ester (PC_71_BM), were purchased from Solarmer Material Inc. (Beijing). Their chemical structures and energy levels are shown in Figure 1. The structure of the PSC device was fabricated by using an inverted configuration, that is, indium tin oxide (ITO) substrate/zinc oxide (ZnO)/photoactive layer/molybdenum oxide (MoO_3_)/silver (Ag). ITO glass was cleaned with deionized water, acetone, chloroform, and isopropyl alcohol in an ultrasonic cleaner, and then, ITO glass needed to be dried in a heat oven. The ZnO electron transport layer was spin-coated (3,000 rpm, 30s) with a colloidal precursor solution (zinc acetate:2-methoxyethanol:ethanolamine = 1 g:10 ml:0.28 ml). After that, the wet ZnO layer was annealed on a hotplate at 200°C for 1 h in air. The active layer precursor solutions, PBDTTT-E (or PTB7):PC_71_BM (10 mg/ml:15 mg/ml), were co-dissolved in chlorobenzene solvent on a 50°C hotplate at the stirring condition of 500 rpm for 12 h, respectively. In addition, to obtain better device performance, about 3% (in volume) 1,8-diiodooctane (DIO) was added to the active layer precursor solution 1 h before spin coating. Afterwards, a 30 µl active layer precursor solution was spin-coated on the ZnO layer with a speed of 1,000 rpm for 60 s. MoO_3_ (8 nm) and Ag electrode (100 nm) layers were sequentially evaporated on the surface of the active layer, and a shadow mask was used to obtain an effective area of the device (0.075 cm^2^) in a vacuum chamber.

### Morphology Characterization and the *J–V* Test

The morphology of PBDTTT-E:PC_71_BM and PTB7:PC_71_BM active layers was characterized by atomic force microscopy (AFM-5500, Agilent) using the tapping mode. The current density–voltage (*J–V*) curve of the device was tested by using an electrochemical workstation (Chenhua, CHI760E) with a linear sweep voltammetry (LSV) method. A light source with an intensity of 100 mW/cm^2^ was corrected by a standard silicon solar cell before testing. The external quantum efficiency (EQE) test of the device was conducted according to the literature (Hu et al., 2021a). Space charge-limited current measurement (SCLC) of PSC devices was performed using previous methods (Shang et al., 2020). All measurements were carried out at room temperature.

### Steady-State Optical Absorption, Photoluminescence, and Spectroelectrochemistry Measurements

The steady-state optical absorption of the active layer was tested on a UV-Vis-NIR spectrometer (Agilent, Cary5000). Photoluminescence measurement of the neat polymer device with the applied bias was carried out previously (Su et al., 2021). The steady-state absorption spectra of polymer cations in the solution and solid state film were obtained by the SEC method. The configuration and operating condition of the SEC measurements were determined using established techniques (Hu et al., 2021b; Hu et al., 2018). In this study, the oxidation potential for the solution and solid state film was applied at 1.5 V to obtain a polymer cation. All of the SEC spectra were obtained from the difference between absorption spectra with and without the oxidation potential. All measurements were carried out in air at room temperature.

### Transient Absorption Measurement

Time-resolved absorption spectroscopy measurements were carried out by using a HARPIA-TA spectroscopy system (HARPIA, light conversion). A fs laser with a pulse width of 190 fs and repetition rate of 100 kHz (1,030 nm, PHAROS, light conversion) was employed as the fundamental laser source of the TA system. The output of the fs laser was divided into two parts. One part was directed into an optical parametric amplifier (OPA, light conversion), and the output of OPA could be used as the pump light. The other part was used to generate probing light, that is, the white light super-continuum (WLSc). The time delay between the pump and probe was adjusted by a mechanical delay stage. All transient absorption measurements were performed at room temperature.

## Results and Discussion

### Photovoltaic Performance of Devices

**FIGURE 2:**
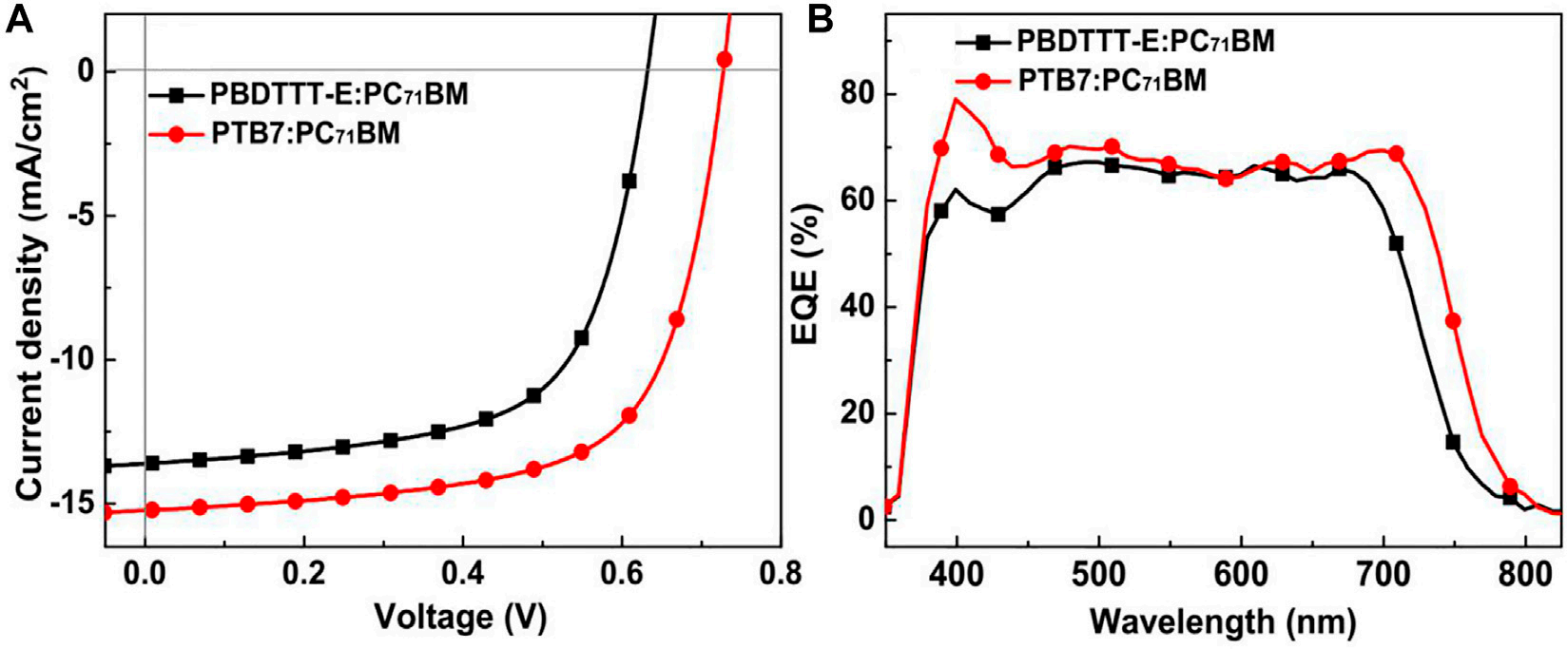
**(A)** Averaged *J–V* curves of PBDTTT-E:PC_71_BM and PTB7:PC_71_BM solar cells. The *J–V* characteristics were averaged from 10 cells. **(B)** EQE curves of PBDTTT-E:PC_71_BM and PTB7:PC_71_BM devices.

**FIGURE 3:**
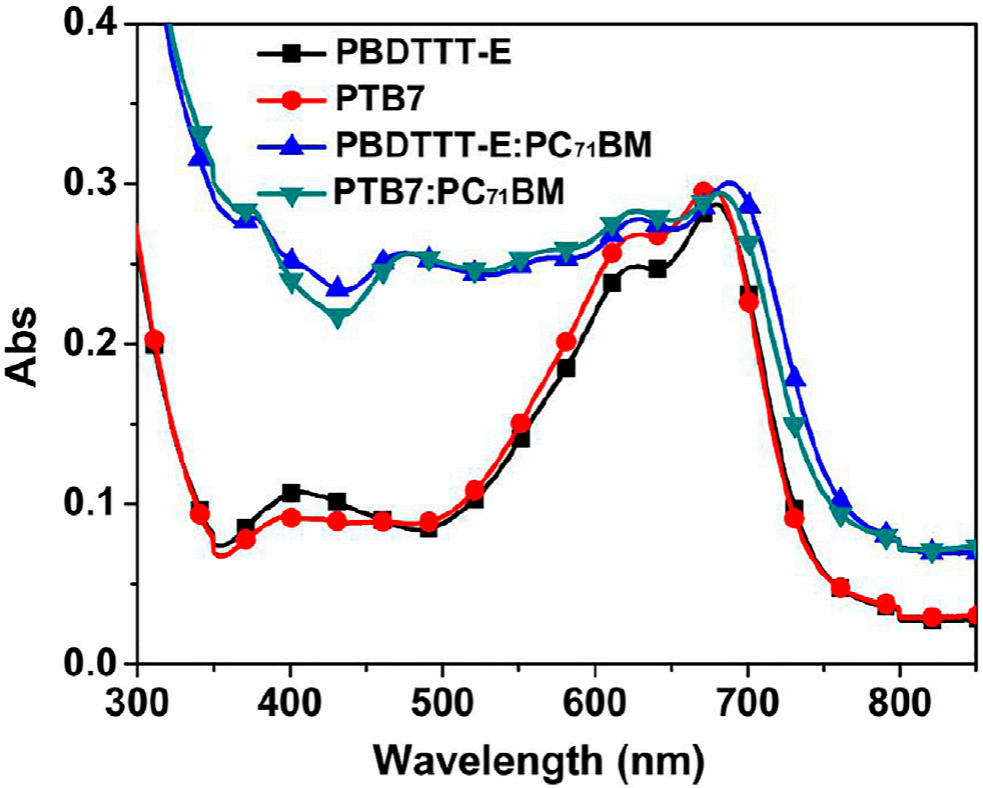
Absorption spectra of PBDTTT-E, PTB7, PBDTTT-E:PC_71_BM, and PTB7:PC_71_BM active layers.

Figure 2 and Table 1 show the *J–V*, EQE characteristic curves, and photovoltaic parameters of PBDTTT-E:PC_71_BM and PTB7:PC_71_BM solar cells, respectively. As for the PBDTTT-E:PC_71_BM solar cell, it shows an open-circuit voltage (*V*_OC_) of 0.632 V, a short-circuit current density (*J*_SC_) of 13.62 mA/cm^2^, and a fill factor (FF) of 64.0%, then achieving an average PCE of 5.51%. Furthermore, these photovoltaic parameters, *V*_OC_, *J*_SC_, FF, and PCE, are improved to 0.727 V, 15.21 mA/cm^2^, 66.6%, and 7.36% in the PTB7:PC_71_BM solar cell, respectively. Obviously, the photoelectric conversion efficiency is enhanced due to the fluorinated TT unit. As for *V*_OC_, the PTB7-based device shows ∼0.1 V higher than that of the PBDTTT-E:PC_71_BM device. It is well known that *V*_OC_ of PSCs is related to the HOMO energy level of the donor and the lowest unoccupied molecular orbital (LUMO) energy level of the acceptor ( $V_{OC}=\frac{1}{\text{e}}\left(\left|E_{HOMO}^{Donor}\right|-\left|E_{LUMO}^{PCBM}\right|\right)-0.3$ ) (Dennler et al., 2008). In this study, the difference between the HOMO of PTB7 and the LUMO of PC_71_BM is ∼0.2 eV larger than that between PBDTTT-E and PC_71_BM (*cf.* Figure 1). Thus, the higher *V*_OC_ of PTB7-based device could be attributed to the lower HOMO of PTB7. To analyse the *J*_SC_ of the two devices, EQE characteristics are depicted in Figure 2B, and the PTB7:PC_71_BM device exhibits a greater EQE characteristic in the wavelength regions of 380–550 nm and 650–800 nm compared with the PBDTTT-E:PC_71_BM device. In the field of PSCs, EQE is usually determined by charge photogeneration and recombination processes; that is, charge photogeneration efficiency is determined by photon absorption efficiency (*ɳ*_a_), exciton diffusion and dissociation efficiency (*ɳ*_ed_), charge transfer and transport efficiency (*ɳ*_ct_), and charge collection efficiency (*ɳ*_cc_), EQE = *ɳ*_a_×*ɳ*_ed_×*ɳ*_ct_×*ɳ*_cc_ (Dang et al., 2013; Lam et al., 2014). Herein, we found that PTB7:PC_71_BM and PBDTTT-E:PC_71_BM films almost had similar absorption characteristics, as shown in Figure 3. This indicates *ɳ*_a_ showing the little difference in two blended films. Besides, their device configurations and fabrication processes are same, that is, ITO/ZnO (30 nm)/active layer/MoO_3_ (8 nm)/Ag (100 nm); thus, the *ɳ*_cc_ should be same. Therefore, the reasonable possibility is the photoelectric conversion difference (*ɳ*_ed_ and *ɳ*_ct_) of two active layers to determine the EQE. FF is an important factor to evaluate the quality of polymer solar cells. Usually, it is the result of the competition between charge recombination (or loss) and charge transport. In this work, the FF of the PTB7-based device (66.6%) was higher than that of the PBDTTT-E:PC_71_BM device (64%), which could be attributed to the less charge loss in the PTB7-based device.

**TABLE 1:** The average photovoltaic parameters of PBDTTT-E:PC_71_BM and PTB7:PC_71_BM solar cells with the corresponding deviations.

Active layers	*V*_OC_ (V)	*J*_SC_ (mA/cm^2^)	FF (%)	PCE (%)
PBDTTT-E:PC_71_BM	0.632 ± 0.005	13.62 ± 0.25	64.0 ± 0.6	5.51
PTB7:PC_71_BM	0.727 ± 0.003	15.21 ± 0.21	66.6 ± 0.7	7.36

### Steady-State Absorption and Photoluminescence Characteristics of Active Layers

To analyze the effect of fluorine substitution on the absorption of active layers, the absorption features of PBDTTT-E, PTB7, PBDTTT-E:PC_71_BM, and PTB7:PC_71_BM active layers were tested by using a spectrometer, as shown in Figure 3. As for the neat PBDTTT-E layer, it exhibits a main absorption band in the wavelength range of 500–800 nm with two absorption peaks at 680 and 626 nm. The former absorption peak is often referred to as the vibronic progression of the electronic state (0′-0), whereas the latter peak is considered as the (1′-0) vibronic absorption of electronic transition, and they both are in relation to the aggregation state of co-polymers (Clark et al., 2009; Hu et al., 2014; Huo et al., 2014; Fauvell et al., 2016). Herein, the relative absorption intensity of the 626 nm peak of the neat PTB7 film (*A*_1´-0_/*A*_0´-0_) is slightly enhanced comparing with that of the PBDTTT-E film, suggesting that the fluorine substituent can influence the co-planar conformation and optical absorption of the polymer (Leclerc et al., 1993; Huo et al., 2013). Comparing with the neat film, the absorption band of the blend film is red-shift, and the absorption intensity is significantly strengthened in the wavelength range of 300–600 nm due to the absorption of PC_71_BM, suggesting that PC_71_BM could also influence the aggregation state and optical absorption of the polymers. As for the blend active layers, PTB7:PC_71_BM and PBDTTT-E:PC_71_BM have similar absorption characteristics in the 300–800 nm region, indicating that *ɳ*_a_ of the two active layers is not the dominant factor to determine the photoelectric conversion performance of devices.

**FIGURE 4:**
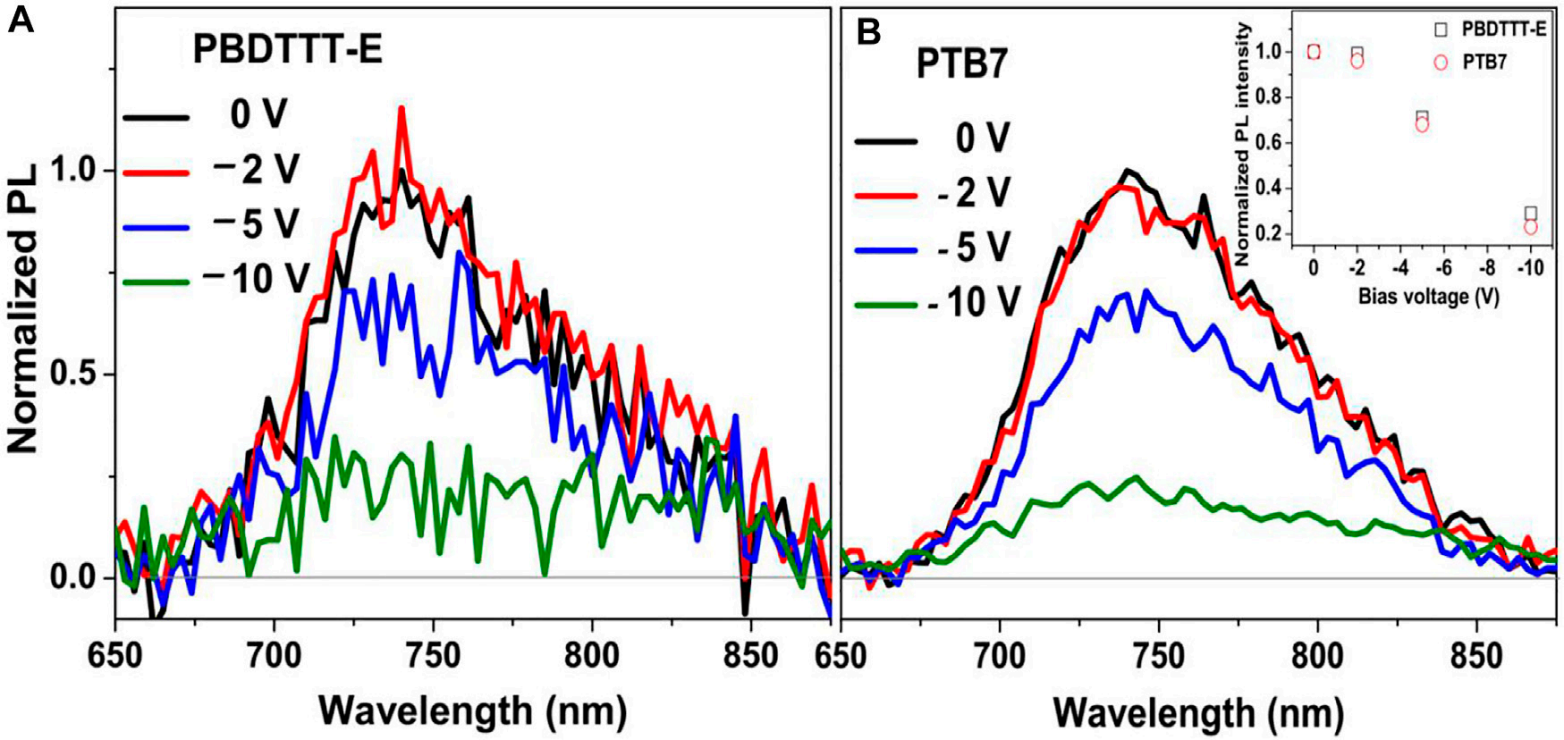
Normalized PL spectra of **(A)** neat PBDTTT-E and **(B)** PTB7 devices after photoexcitation at 532 nm under the various bias electrical field. The inset picture presents the normalized PL intensity of PBDTTT-E and PTB7 devices changed with the bias voltages.

To further study the effect of fluorine substitution on the binding energy of polymer exciton, the PL spectra of neat polymer devices under the various bias electrical field were conducted, as shown in Figure 4. In the field of PSCs, the primary photogenerated species in most of the conjugated polymers is Frenkel-type exciton; thus, “hole–electron” binding energy is usually much higher than room temperature *k*_B_T energy (25 meV, *k*_B_, Boltzmann’s constant, and T, thermodynamic temperature) due to the weak inter-molecular interaction and low dielectric constant (Hedley et al., 2016; Wang et al., 2020). Hence, polymer exciton is hardly dissociated in the neat polymer device at room temperature. Figure 4 shows the PL spectra of neat PBDTTT-E and PTB7 devices after photoexcitation at 532 nm under the different biases. It can be seen that the PL intensity of neat PBDTTT-E and PTB7 devices is unchanged with the external bias range of 0 to −2 V, indicating this electric field has a weak influence on the dissociation of polymer excitons. Interestingly, the PL intensity of the two neat polymer devices is gradually decreased under the high external bias (>−2 V). We notice that the ∼80% PL intensity of the two devices is quenched at a bias of −10 V, indicating 80% of polymer excitons are dissociated by this electric field energy. By using the electric field at −10 V, we estimated exciton binding energies in PBDTTT-E and PTB7 devices are 0.232 and 0.226 eV, respectively (Li H. et al., 2012; Su et al., 2021). Evidently, the neat PTB7 device has similar exciton binding energy with the PBDTTT-E device. Thus, fluorine substitution in PTB7 has a weak influence on exciton binding energy.

### Morphology Characterization of Active Layers

**FIGURE 5:**
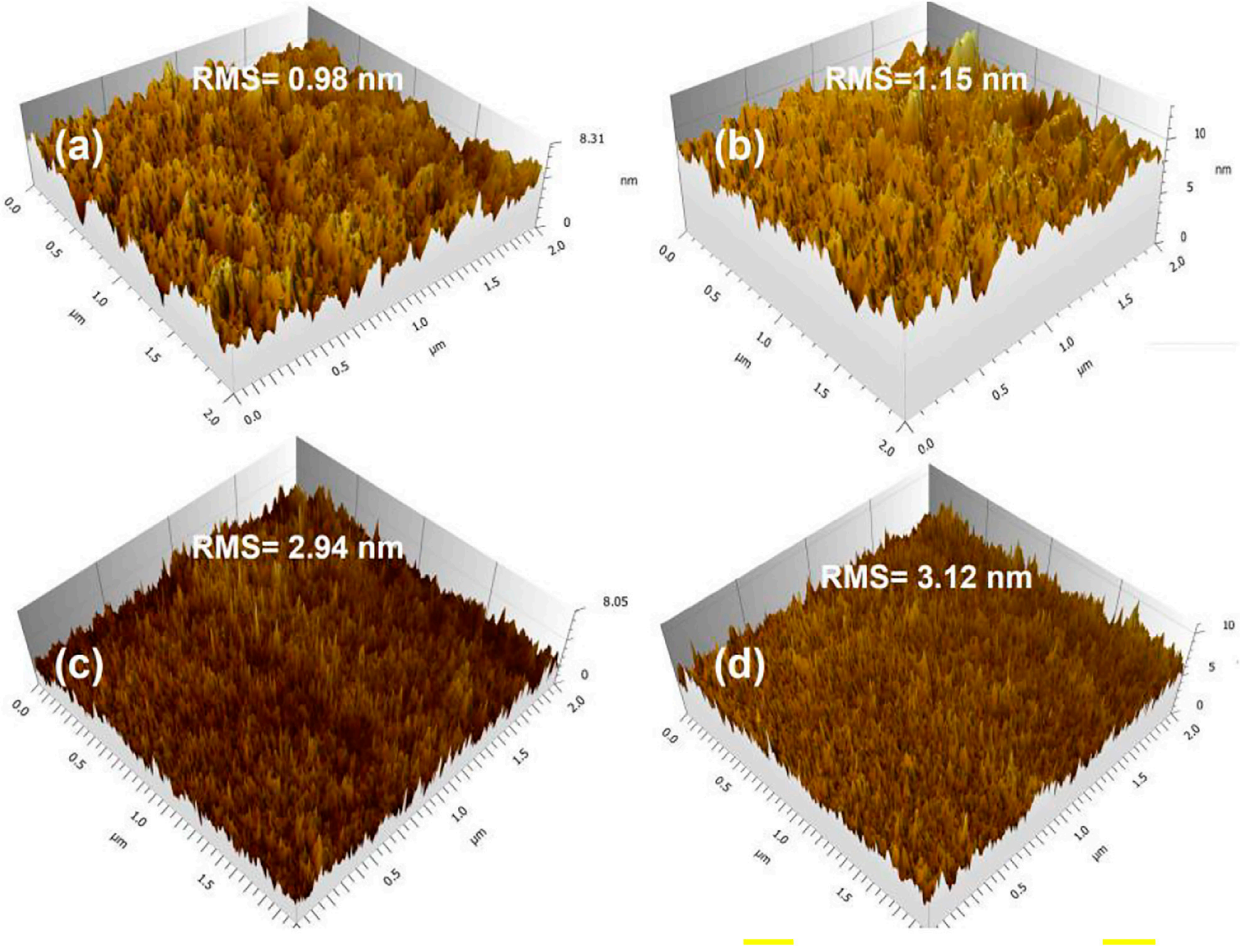
3D AFM topography of **(A)** PBDTTT-E, **(B)** PTB7, **(C)** PBDTTT-E:PC_71_BM, and **(D)** PTB7:PC_71_BM active layers.

To examine the effect of fluorine substitution on the morphology of active layers, the 3D surface features of PBDTTT-E, PTB7, PBDTTT-E:PC_71_BM, and PTB7:PC_71_BM active layers were depicted by using an AFM tester, as shown in Figure 5. Figure 5A shows the surface morphology of the neat PBDTTT-E active layer. It exhibits an overall surface roughness of 0.98 nm (root-mean-square, RMS) in the area of 2 × 2 μm^2^. Figure 5B shows the morphology of the PTB7 active layer. Its surface roughness is measured as 1.15 nm, which is coarser than the PBDTTT-E active layer, indicating that the fluorine-substituted TT unit facilitates the aggregation of the polymer. Figure 5C depicts the morphology of the PBDTTT-E:PC_71_BM active layer; it has a higher roughness (2.94 nm) than the neat PBDTTT-E film. Similarly, the surface roughness of the active layer increases from 1.15 to 3.12 nm when PTB7 was blended with PC_71_BM (Figure 5D). The increased roughness of blend films could be attributed to the formation of donor/acceptor phase separation structures (Zhang et al., 2021). Besides, the PTB7:PC_71_BM layer shows a larger roughness than the PBDTTT-E:PC_71_BM layer, which implies a more pronounced phase separation in the PTB7-based active layer. In PSCs, a blended active layer with appropriate phase separation is expected to facilitate carrier transport and suppress carrier recombination, leading to the efficient PSC device (Guo et al., 2009).

### Transient Absorption and Kinetics Characteristics of PBDTTT-E and PTB7 Films

**FIGURE 6:**
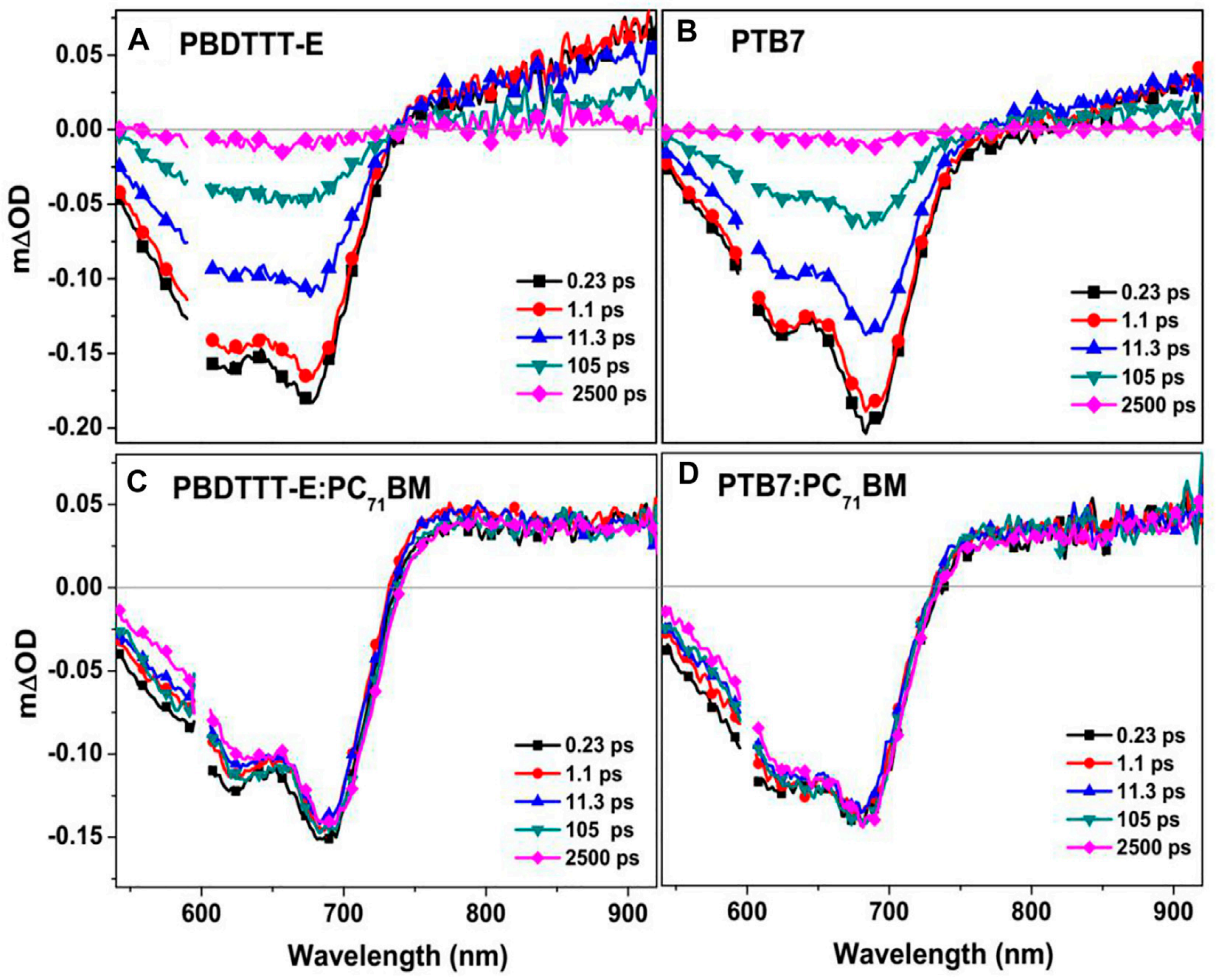
Transient absorption spectra of **(A)** PBDTTT-E, **(B)** PTB7, **(C)** PBDTTT-E:PC_71_BM, and **(D)** PTB7:PC_71_BM films at the different delay times recorded after photoexcitation at 600 nm under an excitation fluency of 2.18 × 10^13^ photons•cm^−2^•pulse^−1^.

In the field of organic solar cells, time-resolved spectroscopy, including transient absorption and transient fluorescence, is a powerful tool for studying the charge photogeneration process (Po et al., 2010; Zhang et al., 2019). In this study, transient absorption spectroscopy was conducted to analyse the excited state and charge photogeneration characteristics from the perspective of molecular structure difference. As seen from Figures 6A,B, PBDTTT-E and PTB7 show similar spectra dynamics in the wavelength range of 550–900 nm in the time range of 0–2.5 ns. Herein, the negative spectra (550–750 nm) are attributed to the bleaching signal. The positive signal band (>750 nm) is the absorption of excited states at different delay times. Figures 6C,D show the transient absorption spectra of PBDTTT-E:PC_71_BM and PTB7:PC_71_BM active layers after photoexcitation at 600 nm (it can excite the polymer phase in the blend film predominately at this excitation energy) under an excitation fluency of 2.18 × 10^13^ photons•cm^−2^•pulse^−1^. Here, the transient absorption spectra of blend active layers are significantly different from those of the neat films. First, the recovery of the ground-state bleaching spectra and the attenuation of the excited-state absorption spectra are much slower. Second, in the near infrared region, the transient absorption spectra feature of blend active layers longer than 1 ps is very similar to the steady-state absorption spectra of polymer cation (PBDTTT-E^•+^ and PTB7^•+^, *cf*. Figure 8). Accordingly, the transient absorption spectrum of the blend films longer than 1 ps is attributed to the spectral dynamics of charge species.

**FIGURE 7:**
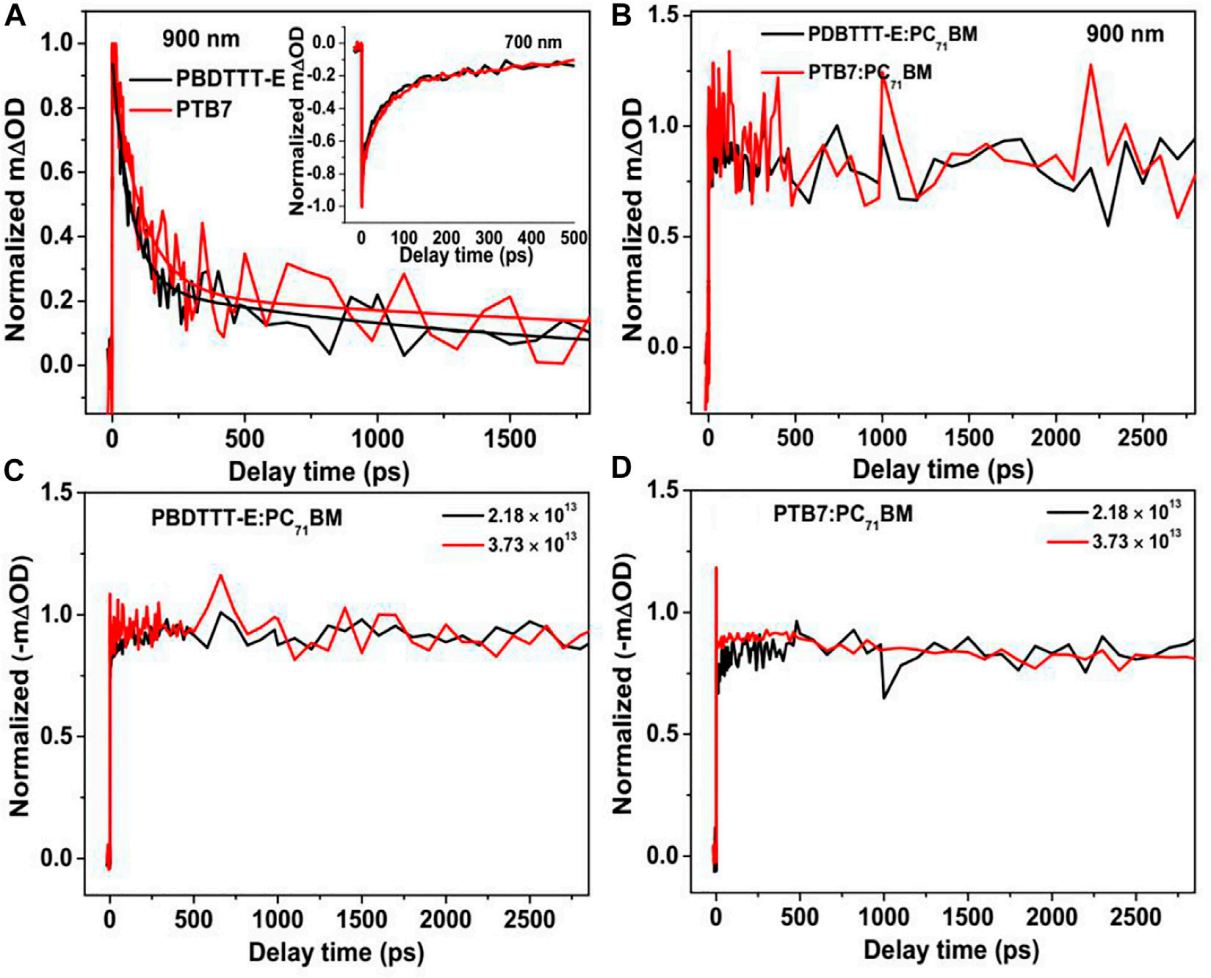
**(A)** Normalized TA kinetics of the neat PBDTTT-E and PTB7 active layers at 900 and 700 nm and **(B)** normalized kinetics of the blend PBDTTT-E:PC_71_BM and PTB7:PC_71_BM active layers at 900 nm; the photoexcitation energy and excitation fluency are 600 nm and 2.18 × 10^13^ photons•cm^−2^•pulse^−1^. Normalized TA bleaching kinetics (700 nm) of the PBDTTT-E:PC_71_BM **(C)** and PTB7:PC_71_BM **(D)** active layer after photoexcitation at 600 nm under the different excitation fluencies (2.18 × 10^13^ and 3.73 × 10^13^ photons•cm^−2^•pulse^−1^).

To understand charge photogeneration dynamics in the active layers, the TA kinetics of neat and blend films need to be analyzed. Figure 7A shows the TA kinetics of neat PBDTTT-E and PTB7 films at 900 nm under the similar excitation condition. It can be seen that the kinetics can be fitted by a bi-exponential decay function, which is a predominated fast decay and a much slower decay in the ns timescale. The fast decay components of PBDTTT-E and PTB7 films are 69.6 ± 5.4 ps and 108.1 ± 11.6 ps, respectively. The fast decay component could be attributed to the decay of excitons in neat polymer films (Su et al., 2021). The slower decay component in PBDTTT-E and PTB7 films exhibits a long lifetime of 1,598 ± 386 ps and 2,292 ± 1,140 ps, which could be attributed to the decay of photogenerated charge (Guo et al., 2009; Guo et al., 2010). We note that the exciton lifetime of PTB7 is longer than that of PBDTTT-E. Assuming the exciton diffusion coefficients of these two neat films are comparable, a longer exciton lifetime would result in a longer exciton diffusion length, which is a critical factor for enhancing the exciton dissociation efficiency in blend films.

For the kinetics in the blend films after photoexcitation polymers predominately at 900 nm, we note that all the kinetics decays are very slow, as shown in Figure 7B. Interestingly, no fast decay corresponding to the exciton dissociation of the polymer is observed in the kinetics, indicating the exciton diffusion and dissociation processes happen within the instrumental response function of 200 fs. In polymer solar cells, the size of the donor phase can determine the exciton diffusion coefficient and exciton lifetime in the blend film. The short exciton lifetime in the blend films suggests all the photogenerated excitons can arrive at the D/A interface very quick. In other words, the size of the donor phase is very small in both PBDTTT-E:PC71BM and PTB7:PC71BM active layers.

Geminate recombination and non-geminate (or bimolecular) recombination are two channels of charge loss during the process of the free charge transport in polymer solar cells. Herein, in order to clarify the charge recombination characteristics in PBDTTT-E:PC_71_BM and PTB7:PC_71_BM active layers, the 600 nm excitation energy with varying excitation fluencies (2.18 × 10^13^ and 3.73 × 10^13^ photons•cm^−2^•pulse^−1^) was used to excite the active layers, as shown in Figures 7C,D. It can be seen that both PBDTTT-E:PC_71_BM and PTB7:PC_71_BM active layers exhibit similar decay behaviors in the time range of 0–3 ns under the low and high excitation fluencies. Hence, it can be inferred that the non-geminate recombination process is negligible at the ultrafast timescale. Based on this, we speculate that the difference of charge loss between PBDTTT-E:PC_71_BM and PTB7:PC_71_BM may originate from the bimolecular recombination process at a longer timescale (>3 ns). Besides, the kinetics shows a slow-rise process at the early timescale in Figures 7C,D. By examining TA spectra at varying delay time (Figures 6C,D), we find that the bleaching is red-shifting with delay time, which can be attributed to the transport of free carriers from high-energy states to lower-energy states. Herein, the detection wavelength of 700 nm is on the side of the red-shift of the bleaching peak, and thus, the kinetics at 700 nm would show a slow rise with the shift of the bleaching peak. Meanwhile, we also note that the rise time of the kinetics in Figures 7C,D is comparable to the red-shift time of the bleaching peak. Therefore, the rise process in the kinetics could be attributed to that the charge transport induced the shifting of bleaching peaks in TA spectra.

### Spectral Characterization of the PBDTTT-E and PTB7 Radical Cations in Solution, Neat, and Blended Films

The generation and transport of charge species are very important for the photocurrent of polymer solar cells. Polymer-positive polaron and -negative polaron could be generated by the photoexcitation process, and their transient absorption features usually have a P1 band and P2 band in the near infrared region, corresponding to the electron transition from HOMO to SOMO and SOMO to LUMO energy levels, respectively (Österbacka et al., 2000; Jiang et al., 2003). In this contribution, to analyze the effect of fluorine substitution on the charge transport in active layers, we employed an electrochemical method to produce PBDTTT-E^•+^ and PTB7^•+^ cations under the oxidation potential. Note that the steady-state absorption spectra of the polymer cation and transient absorption spectra of polymer transient polarons should be similar under the electrochemical and photoinduction methods.

**FIGURE 8:**
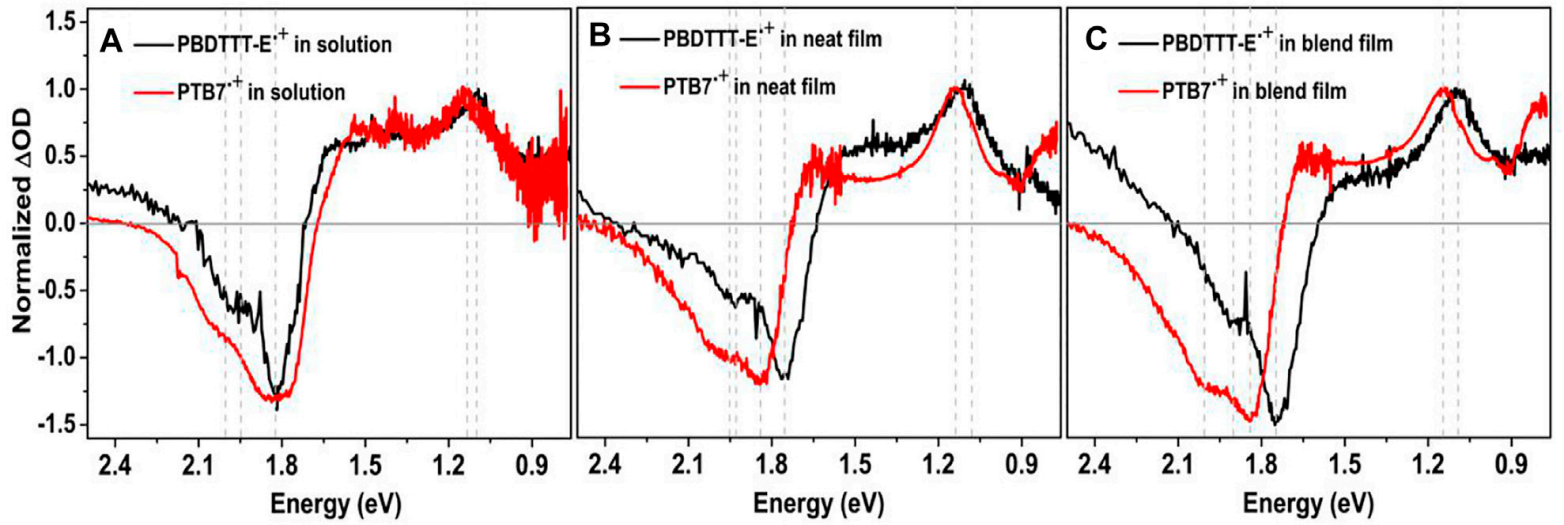
**(A-C)** Spectroelectrochemical spectra of PBDTTT-E, PTB7, PBDTTT-E:PC_71_BM, and PTB7:PC_71_BM in solution and films. The applied oxidation bias was controlled at 1.5 V.

Figure 8A shows the characteristic absorption spectra of PBDTTT-E^•+^ and PTB7^•+^ in solution. The negative spectra band at 1.67–2.36 eV is designated as the electrobleaching signal that is induced by the oxidation depletion of a polymer molecule under the applied potential. Meanwhile, the positive absorption spectra appear in the near infrared region, which can be attributed to the absorption of the polymer radical cation. The positive absorption spectra and negative electrobleaching suggest a correlation between the generation process of the polymer radical cation and the oxidation depletion of the polymer (Hu et al., 2021a). As for PBDTTT-E^•+^ in solution, it shows a P2 absorption band, corresponding to the transition between SOMO to LUMO energy levels, in the region of 0.9–1.6 eV with an absorption peak at 1.09 eV (Table 2). However, this absorption band shows a slight blue shift for PTB7^•+^, and the P2 peak changes to 1.14 eV, about 0.05 eV blue shift compared to PBDTTT-E^•+^. Assuming that the LUMO levels of PBDTTT-E^•+^ and PTB7^•+^ cations are similar, the blue shift of the P2 band would suggest a lower energy level of SOMO in PTB7^•+^. In neat films, we also observe a similar blue-shift characteristic for the P2 peak with ∼0.04 eV after PBDTTT-E via fluorine substitution.

**TABLE 2:** Transition energies (in eV) of the characteristic spectral features of polymer cations in the solution and neat and blend active layer.

State	PBDTTT-E cations (peaks)			PTB7 cations (peaks)		
	Bleaching	P2	P1	Bleaching	P2	P1
Solution	1.82, 1.96	1.09	—	1.82, 2.02	1.14	—
Neat film	1.75, 1.93	1.10	—	1.84, 1.95	1.14	∼0.79
Blend film	1.75, 1.90	1.09	—	1.84, 1.99	1.15	∼0.79

Furthermore, we analyzed the difference of PBDTTT-E^•+^ and PTB7^•+^ in blend active layers, as seen from Figure 8C and Table 2. In the blend films, the electrobleaching spectrum and P2 peak of PTB7^•+^ are both blue shift compared with PBDTTT-E^•+^ (1.75–1.84 eV, 1.90–1.99 eV, and 1.09–1.15 eV). The blue-shift feature in the blend active layer is even more pronounced than that in the solution and neat polymer films. For PBDTTT-E^•+^, we find the P2 peak energy in the neat film is ∼0.01 eV higher than that in the blend film, while the P2 peak energy in the neat film is ∼0.01 eV lower than that in the blend film for PTB7^•+^. As mentioned above, a higher P2 energy would suggest a lower SOMO level. Here, we deduce that the SOMO level energy of PTB7^•+^ in the blend film is lower than that in the neat PTB7 film. On the contrary, the SOMO level energy of PBDTTT-E^•+^ in the blend film is higher than that in the neat film. In polymer solar cells, there are two situations for the donor phase in the blend film: one is the polymers at the D/A interface, and the other is the polymers in the center of the donor phase which is similar to that in the neat polymer film. Considering these two situations in blend films, we deduce the SOMO energy level of PTB7^•+^ in the center of the donor phase is higher than that at the interface. This energy level distribution could facilitate the hole transport from the interface into the center of the donor phase and suppress the charge recombination processes. On the contrary, for the PBDTTT-E blend film, the energy level distribution is not conducive to the transport of holes at the interface.

### Electrical Characteristics of Devices

**FIGURE 9:**
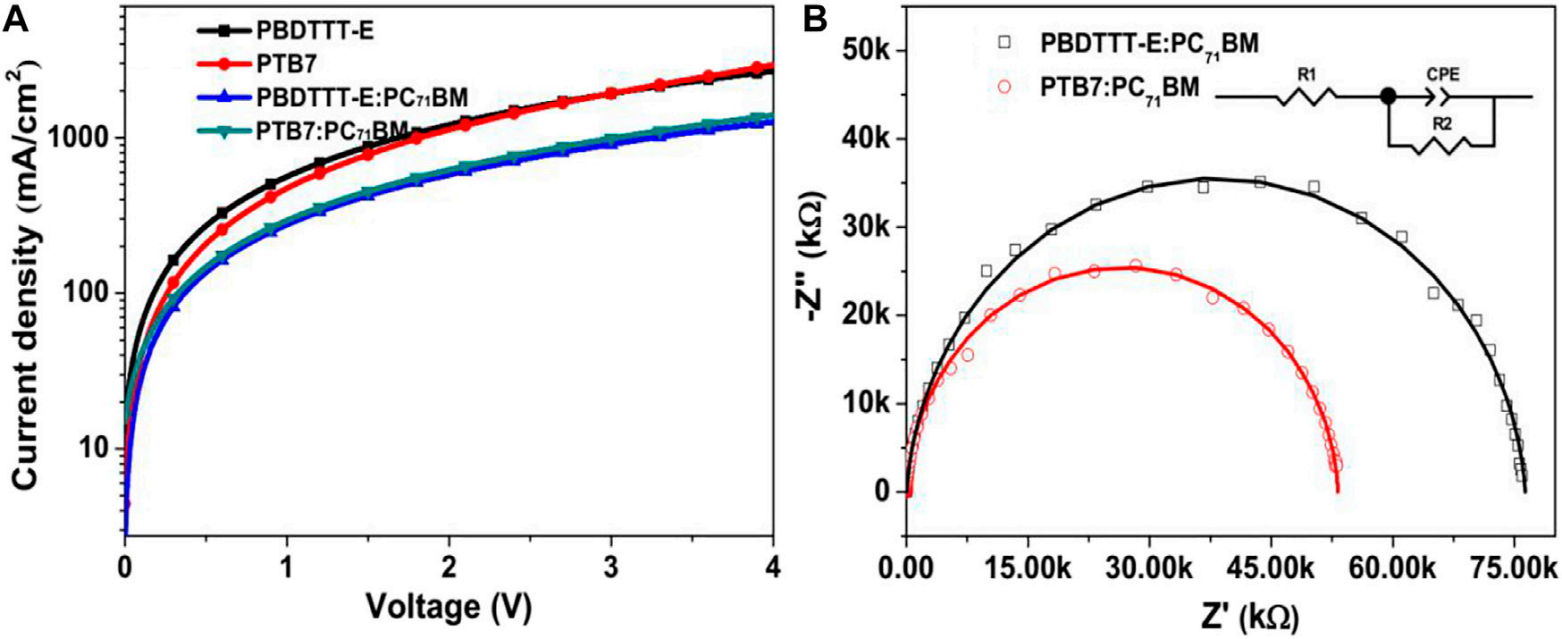
**(A)** *J–V* characteristics of hole-only devices based on the configuration of ITO/MoO_3_/active layer/MoO_3_/Ag. **(B)** EIS spectra of PBDTTT-E:PC_71_BM and PTB7:PC_71_BM devices.

To further quantitatively analyze the charge transport property in PSCs, SCLC measurement was carried out to estimate the charge carrier mobility according to the literature (Zhang et al., 2014, and the *J–V* curves are shown in Figure 9A. Hole mobility (*μ*_h_) can be calculated using the Mott–Gurney law follows:

$$J=\frac{9}{8}\varepsilon_{0}\varepsilon_{\text{r}}\mu_{\text{h}}\frac{V^{2}}{d^{3}}.$$ (1)

Here, *ε*_r_ is the relative permittivity of the conjugate polymer (*ε*_r_ = 3), *ε*_0_ is the vacuum dielectric constant (8.85 × 10^−12^ F m^−1^), *V* is the applied bias, and *d* is the thickness of the active layer. We obtained the thicknesses of neat films and blend films as ∼90 and ∼110 nm. By calculation, the hole mobilities are 4.1 × 10^−3^, 4.5 × 10^−3^, 3.1 × 10^−4^, and 3.4 × 10^−4^ cm^2^ V^−1^ s^−1^ in PBDTTT-E, PTB7, PBDTTT-E:PC_71_BM, and PTB7:PC_71_BM devices, respectively. Apparently, PTB7-based devices have higher hole mobilities than PBDTTT-E-based devices, suggesting that fluorine substitution has an important effect on transport efficiency (*ɳ*_ct_). To further analyse charge carrier recombination characteristics in blend devices, EIS measurements of devices were conducted under the dark condition, as shown in Figure 9B. The results of the two blend devices were analysed with an equivalent circuit (inset picture of Figure 9B). Herein, R1 contains the electrodes, the active layer/metal electrode interface, and the active layer. The constant-phase element (CPE) is usually used to simulate the dielectric effect of the device for better fitting. R2 is often considered as a charge-transfer (or recombination) resistor. Thus, it reflects the charge carrier recombination. In this work, all EIS spectra were fitted by a simulation function installed in the measurement software (Zview2), and the fitting parameters are shown in Table 3. R1 values are 38.57 and 23.36 Ω in PBDTTT-E:PC_71_BM and PTB7:PC_71_BM devices, respectively, indicating that the PTB7:PC_71_BM device has a small photocurrent loss at the active layer or interface. Besides, the PTB7:PC_71_BM device shows a smaller R2 value (53,200 Ω) than the PBDTTT-E:PC_71_BM device (76,286 Ω). Hence, it implies that the PTB7-based device is more favorable for suppressing charge recombination during the charge transport process. CPE values also indicate a better charge transport channel in the PTB7:PC_71_BM device.

**TABLE 3:** Electrical parameter values in blend devices.

Devices	R1 (Ω)	R2 (Ω)	CPE-T (F)	CPE-P
PBDTTT-E:PC_71_BM	38.57	76,286	2.48 × 10^−9^	0.9468
PTB7:PC_71_BM	23.36	53,200	2.75 × 10^−9^	0.9719

## Conclusion

In summary, we have spectroscopically characterized the exciton and polaron species in the neat PBDTTT-E (PTB7) and the blend PBDTTT-E (PTB7):PC71BM active layers and investigated their charge transport and charge recombination behaviors. PTB7 and PBDTTT-E showed a fluorine atom substitution difference on the TT unit, leading to the different device performance. Our results showed that the influence of fluorine substitution on the exciton binding energy of the polymer and the carrier recombination dynamics in the ultrafast timescale is insignificant. Besides, we found that the fluorine substitution in the conjugated polymer could enhance the exciton lifetime in the neat polymer film, and it also could increase the mobility of photogenerated charge. Moreover, we found that the SOMO energy level distribution of the donor in PTB7:PC71BM solar cells could facilitate hole transport from the D/A interface to the center of the donor phase, showing a better advantage than the PBDTTT-E:PC71BM solar cell. Longer exciton lifetime and appropriate energy level arrangement played critical roles for an efficient PTB7:PC71BM device.

## Data Availability

The original contributions presented in the study are included in the article/supplementary material, further inquiries can be directed to the corresponding authors.
